# Effects of Perchlorate and Other Groundwater Inorganic Co-Contaminants on Aerobic RDX Degradation

**DOI:** 10.3390/microorganisms10030663

**Published:** 2022-03-20

**Authors:** Amit Yadav, Swati Gupta, Paula Istvan, Zeev Ronen

**Affiliations:** 1 Department of Environmental Hydrology and Microbiology, The Zuckerberg Institute for Water Research, The Jacob Blaustein Institutes for Desert Research, The Ben-Gurion University of the Negev, Sde Boker Campus, Midreshet Ben-Gurion, Beersheba 849900, Israel; yadava@post.bgu.ac.il (A.Y.); swati@iitk.ac.in (S.G.); paula.istvan@mn.uio.no (P.I.); 2 Department of Chemistry, Indian Institute of Technology Kanpur, Kanpur 208016, Uttar Pradesh, India; 3 Center of Bioinformatics, University of Oslo and Cancer Registry, N-0304 Oslo, Norway

**Keywords:** RDX, biodegradation, perchlorate, chlorate, *xplA*

## Abstract

Hexahydro-1,3,5-trinitro-1,3,5-triazine (RDX) pollution is accompanied by other co-contaminants, such as perchlorate and chlorates, which can retard biodegradation. The effects of perchlorate and chlorate on aerobic RDX degradation remain unclear. We hypothesized that they have a negative or no impact on aerobic RDX-degrading bacteria. We used three aerobic RDX-degrading strains—*Rhodococcus* strains YH1 and T7 and *Gordonia* YY1—to examine this hypothesis. The strains were exposed to perchlorate, chlorate, and nitrate as single components or in a mixture. Their growth, degradation activity, and gene expression were monitored. Strain-specific responses to the co-contaminants were observed: enhanced growth of strain YH1 and inhibition of strain T7. Vmax and Km of cytochrome P450 (XplA) in the presence of the co-contaminants were not significantly different from the control, suggesting no direct influence on cytochrome P450. Surprisingly, *xplA* expression increased fourfold in cultures pre-grown on RDX and, after washing, transferred to a medium containing only perchlorate. This culture did not grow, but *xplA* was translated and active, albeit at lower levels than in the control. We explained this observation as being due to nitrogen limitation in the culture and not due to perchlorate induction. Our results suggest that the aerobic strain YH1 is effective for aerobic remediation of RDX in groundwater.

## 1. Introduction

Hexahydro-1,3,5-trinitro-1,3,5-triazine (RDX) is a widely used explosive in the military and construction industries [1]. RDX is characterized by relatively high thermal stability, high density, and high detonation velocity, all of which promote its extensive use [2,3]. Over the past century, RDX has become a significant global pollutant, and there are mounting concerns over its toxicity to biological and environmental systems. The manufacturing, loading, assembly, and packaging of RDX into munitions for use in frontline research, at training sites, and on the battlefield have resulted in terrestrial and aquatic pollution [4]. Consequently, some sites have been reported to contain explosives and associated compounds at concentrations that threaten the environment and human health [5]. One such site is Israel’s coastal aquifer below manufacturing facilities, which is contaminated with concentrations of up to 2 mg/L RDX [6], 1200 mg/L perchlorate, 268 mg/L chlorate, 204 mg/L, and nitrate [7,8]. Groundwater supply to surrounding municipalities has been halted because of the pollution. Thus, there is an urgent need to remove the hazardous RDX to ensure public safety and protect natural resources, especially in a semiarid environment such as Israel.

RDX can be biodegraded under both anaerobic and aerobic conditions through various pathways [9]: reductive, anaerobic, and aerobic ring cleavage leading to mineralization. Under anaerobic conditions, subsequent reduction involves two-electron transfer of the -N-NO_2_ functional groups, giving the corresponding nitroso derivatives, such as MNX, DNX, and TNX, (mono-, di-, and tri-nitroso derivatives, respectively), which are more toxic [10]. On the other hand, aerobic denitration involves cleaving the -N-NO_2_ bonds, leading to the formation of 4-nitro-2,4-diazabutanal (NDAB), nitrite, ammonia, formaldehyde, and formic acid [11]. Several strains of the genus *Rhodococcus*, such as DN22 from Australia [12], 11Y from England [13], and YH1 [14], T7, and T9N from Israel [15], have been identified as RDX degraders via these steps. The denitration mechanism was found to be catalyzed by a unique form of the enzyme cytochrome P450, encoded by the gene *xplA*, with NADPH as an electron donor and resulting in NDAB as a stable, non-toxic product [16]. Thus, the biodegradation capacity of RDX by different bacteria can be used to treat polluted groundwater.

Because RDX is used as a nitrogen source, it is expected that external inorganic nitrogen compounds will affect *xplA* expression. For instance, in *Gordonia* sp. strain KTR9, nitrogen availability, as ammonium or nitrate, is a significant determinant of RDX degradation and *xplA* expression [17]. Although deletion of *glnR* (encoding a regulatory protein affecting nitrogen assimilation in diverse *Actinobacteria*) also abolished the inhibition of *xplA* expression by nitrite, there was no evidence for a direct role of *glnR* in regulating *xplA* expression. Instead, the general availability of nitrogen repressed *xplA* expression [18]. Similarly, in *Rhodococcus rhodochrous* 11Y, *xplA* expression was induced under nitrogen-limiting conditions and further enhanced by the presence of RDX [19].

Groundwater contaminated with explosives and propellants (perchlorate, for example) is often treated in an aboveground bioreactor, especially when pumping can control fast-migrating pollutants. The advantage of this method is the ability to precisely control biodegradation by maintaining physical and hydrochemical conditions, and the production of high-quality effluents. However, when inorganic co-contaminants are present together with RDX in the bioreactor, they can interfere with each other’s removal, as demonstrated in anaerobic fluidized bed reactors [20]. In another example, nitrate, RDX, and perchlorate were effectively reduced in microcosms and flow-through columns using a vegetable oil substrate. Nitrate and perchlorate were rapidly biodegraded, followed by RDX and HMX, implying their inhibitory effect [21]. A later field experiment showed that a bio-barrier containing vegetable oil substrate in a polluted shallow aquifer removed both explosives and perchlorate. However, the toxic intermediate(s) of anaerobic degradation of RDX and metals such as arsenic were observed in the downgradient treated water, emphasizing the limitation of the anaerobic remediation approach [22].

The potential effect of inorganic co-contaminants in the groundwater on RDX degradation depends on the redox conditions—aerobic or anaerobic. The co-contaminants can compete with RDX as substrate and may have a toxic effect on the degrading organisms. Under aerobic conditions, nitrate can serve as an alternative nitrogen source for the RDX-degrading bacteria [23,24]; however, the effects of perchlorate and chlorate are not known. Under anaerobic conditions, perchlorate, chlorate, or nitrate can serve as favored electron acceptors [22]. Nitrate can also serve as an alternative to nitrogen. Perchlorate or chlorate (strong oxidizers) can induce oxidative stress in the cells and thus slow down degradation [25]. It has been shown that in the genomes of perchlorate-reducing bacteria, the perchlorate reduction genomic island always includes oxidative stress genes, such as *rpoS*, which indicates the oxidative damage caused by perchlorate [26].

Because the aerobic degradation of RDX does not produce toxic intermediates, the use of RDX-degrading strains, such as *Gordonia* KTR9, has been tested for the bioremediation of polluted water [27,28,29]. Those studies showed that in situ bioaugmentation and biostimulation of aerobic bacteria lead to rapid degradation of RDX in a polluted aquifer over a long period [30]. In Israel, in contrast, the planned remediation of the coastal aquifer is via the “pump and treat” method to control the migration of perchlorate and chlorate [31], where six production wells are expected to supply 650 m^3^/h each to a biological treatment plant. This provides an opportunity to design an aerobic–anaerobic system to treat RDX first aerobically, and then the rest of the contaminants in an anaerobic stage. However, the fundamental question of how aerobic RDX degradation is affected by co-contaminants has never been addressed.

We hypothesized that during ex situ (pump and treat) bioremediation of polluted groundwater, the occurrence of toxic co-contaminants will affect the efficiency of aerobic RDX degradation by *Rhodococcus* strains in bioreactors treating contaminated groundwater. Our research objectives were first, to test and compare the effects of the co-contaminants on the RDX degraders’ activity and growth. These degraders had been isolated from different environments, such as the Israeli coastal aquifer, and included *Rhodococcus* strains YH1 [14] and T7 [15], and *Gordonia* strain YY1 [32]. Then, we examined the effects of the co-contaminants (individually or as a mixture) on the intrinsic degradation activity of strain YH1. Finally, we tested whether the co-contaminants affect *xplA* expression in strain YH1. Our results are expected to contribute to the better design of an ex-situ bioreactor for treating groundwater containing RDX and co-contaminants in Israel using the effective RDX degraders—*Rhodococcus* strains.

## 2. Materials and Methods

### 2.1. Chemicals, Reagents, and Strains

RDX, purity 99%, was obtained from Israel Military Industries (IMI Systems). All chemicals used in the experiment were of high-performance liquid chromatography (HPLC) and analytical grade. Bacterial strains were YH1 [14], T7 [15], and YY1 [31] isolated from polluted soil (YH1 and YY1) and contaminated groundwater (T7). Stocks of all strains were preserved in glycerol at −80 °C.

### 2.2. Growth Experiments with Strains YH1, T7 and YY1

Minimal medium was prepared by combining K_2_HPO_4_ (1.5 g/L), KH_2_PO_4_ (0.5 g/L), and 10 mL trace elements. The trace elements consisted of (g/L) Na_2_EDTA (12), FeSO_4_7H_2_O (2), CaCl_2_2H_2_O (1.32), Na_2_SO_4_ (10), ZnSO_4_7H_2_O (0.4), MnSO_4_H_2_O (0.3), CuSO_4_5H_2_O (0.1), NaMoO_4_2H_2_O (0.1), and 0.5 mL H_2_SO_4_ in 1 L of double-distilled water (DDW). The pH of the medium was adjusted to 7.2–7.4. The solutions were autoclaved and cooled. RDX (20 mg/mL stock solution) was first dissolved in acetone and then added into an empty sterilized bottle to a final concentration of 20 mg/L. The acetone was evaporated and minimal medium was added, and then stirred in the dark overnight. Once RDX had dissolved in the minimal medium, a sterile solution of MgSO_4_7H_2_O was added to a final concentration of 200 mg/L. Then, glucose or other carbon sources were added to the desired concentration (1000 mg/L) from filtered sterilized stock solutions.

### 2.3. Bacterial Inoculum Preparation and Growth Conditions

Strains were retrieved from −80 °C glycerol stocks and first grown in RDX-containing agar (1.5%) medium on plates. Then, a single colony was picked after 48–96 h and inoculated into 50 mL minimal medium containing 20 mg/L RDX. Cultures were grown at 30 °C on a rotary shaker at 150 rpm. Bacterial growth was measured as optical density at 600 nm (OD_600_). Cells in late log phase (~48 h) were then pelleted by centrifugation at 10,000 g for 5 min at 4 °C washed twice with sterile minimal medium to remove residual glucose.

### 2.4. Experimental Setup for Growth with Co-Contaminants (Perchlorate, Chlorate, Nitrate and Their Mixture) and RDX

Growth experiments were set up to measure the ability of *Rhodococcus* strains YH1, T7, and YY1 to utilize RDX as sole nitrogen source in the presence of external co-contaminant sources. For each strain, experiments were set up in 250 mL conical flasks containing 50 mL minimal medium supplemented with 20 mg/L RDX. The setup was divided into four treatments: perchlorate (800 mg/L), chlorate (200 mg/L), nitrate (60 mg/L), and their mixture, representing external co-contamination. The co-contaminants’ concentrations were based on their actual recently measured contamination in Israel’s groundwater [8]. All treatments were carried out in 3 biological replications, and the initial OD_600_ was 0.05. The flasks were incubated at 30 °C with shaking for up to 96 h. Samples were taken every 12 h to determine growth (OD_600_), then centrifuged and their supernatant collected to measure RDX levels by HPLC (Section 2.7.2). Data were analyzed using GraphPad Prism (version 9 for Windows, GraphPad Software, San Diego, CA, USA, www.graphpad.com, accessed on 12 November 2021). A nonlinear exponential model was used to extract the growth rate from the log phase of the growth curve. Two-way repeat measures ANOVA was used to assess the differences between the curves for each treatment by Bonferroni method post-test.

The critical dose response effect of the different contaminants was determined using nitrate, perchlorate, and chlorate for strains YH1, T7, and YY1. Bacteria were grown at 30 °C in minimal medium containing 20 mg/L RDX. The experimental setup contained minimal medium with RDX and varying concentrations of KNO_3_ and NaClO_4_ and NaClO_3_. The salinity effect was tested with the same concentrations of NaCl. Minimal medium supplemented with 20 mg/L RDX and glucose served as a positive control; initial OD_600_ was 0.05. All treatments, including positive controls, were carried out in 3 biological replications. The flasks were incubated at 30 °C with shaking for up to 96 h (New Brunswick Scientific Co. G25 at 150 rpm, Enfield, CT, USA). Then, samples were taken, growth was determined by OD_600_, samples were centrifuged and their supernatant collected, and RDX levels were measured by HPLC (Section 2.7.2).

### 2.5. Resting Cell Kinetics with Co-Contaminants (Perchlorate and Chlorate) and RDX

Cultures were grown at 30 °C in minimal medium (with perchlorate or chlorate), and 1000 mg/L glucose was added. At the late log phase (48 h), cells were harvested, washed twice in minimal medium, and resuspended. Experiments were set up in 250 mL conical flasks containing 25 mL minimal medium supplemented with RDX (1–20 mg/L). The setup was divided into three treatments: perchlorate (800 mg/L), chlorate (200 mg/L), and a control. Cells (0.05 at OD_600_) were incubated in minimal medium without carbon for 3 h. The positive control consisted of culture suspension pre-grown on RDX alone. Samples were taken at 1 h intervals, centrifuged at 10,000× *g* for 5 min at 4 °C, and the supernatant was used for RDX analysis by HPLC (Section 2.7.2). We used GraphPad Prism 9 to calculate Vmax (maximum rate of reaction) and Km (Michaelis constant) with the nonlinear regression fitting Michaelis–Menten model. Two-way ANOVA was used to test the treatment effects on Km and Vmax.

### 2.6. Expression of xplA in Strain YH1

For the expression experiment, strain YH1 was first grown in minimal medium containing RDX (20 mg/L). Cells were then incubated with RDX (20 mg/L) and perchlorate (800 mg/L), or with perchlorate alone. The control treatments consisted of incubation of YH1 with perchlorate and ammonium (5 mM) or perchlorate and potassium nitrate (5 mM). Additional controls consisted of cultures grown with only ammonium and RDX. All treatments were carried out in 3 biological replications, and the initial OD_600_ was 0.05. Flasks were incubated at 30 °C with shaking for up to 96 h. Every 24 h, samples were taken and bacterial growth was determined. The collected samples were centrifuged (6000 g for 5 min at 4 °C), the pellet was collected to determine XplA activity and for RNA extraction (stored at −20 °C; see Section 2.8), and the supernatant was used for RDX measurement by HPLC (Section 2.7.2).

For treatments without RDX (perchlorate only, perchlorate with ammonium, perchlorate with potassium nitrate, and ammonium only), at 24 h, the pellet was resuspended in minimal medium containing 20 mg/L RDX without a carbon source. Suspensions were incubated at 30 °C with shaking for up to 3 h, and samples were harvested at 1 h intervals. Growth and RDX degradation were determined. The instantaneous degradation rate was calculated according to the slope of the linear curve fitting the RDX concentrations.

### 2.7. Analytical Techniques

#### 2.7.1. Spectrophotometry

Bacterial growth was measured at 600 nm using an Infinite^®^ 200 PRO (Tecan, Männedorf, Switzerland).

#### 2.7.2. HPLC

HPLC measurements were performed with an Agilent 1100 HPLC instrument (Waldbronn, Germany) equipped with a DAD (Diode-Array Detection) detector. Samples, 25 μL, were separated on a Luna C18 column (Phenomenex, Torrance, CA, USA), and the mobile phase consisted of 50% methanol and 50% water, with a flow rate of 1 mL/min. RDX in filtered (0.22 μm) culture supernatant was detected and quantified at 254 nm. The RDX peak was identified by comparing the retention time with an authentic RDX standard. Standard curves of concentration versus peak area were constructed for every run of samples and converted peak area data of samples to concentrations of RDX. The calibration curve was linear at a concentration of 0.1 to 20 mg/L.

#### 2.7.3. Dissolved Organic Carbon (DOC) and Dissolved Nitrogen (DN)

Culture samples were filtered and then diluted with DDW for DOC and DN analyses. Samples were analyzed by TOC/TN analyzer (multi N/C 2000S, Analytik Jena, Jena, Germany).

### 2.8. RNA Extraction from Bacterial Isolates

Total RNA was isolated from 200 mL bacterial culture (strain YH1) grown in minimal medium containing RDX (20 mg/L) using Total Nucleic Acids Extraction from Soil V.2 method as described in [33]. Bacterial cells were harvested by centrifugation at 6000× *g* for 5 min at 4 °C. Then, 375 µL phosphate buffer (120 mM), 125 µL TNC (Trizma, NaCl, and CTAB), and 400 µL TE-saturated phenol were added and the sample was homogenized at 4 °C. The homogenate was centrifuged at 14,000 g for 3 min, and the supernatant was transferred to another fresh tube, to which one volume of phenol/chloroform/isoamyl alcohol (25:24:1, *v*/*v*) was added. The tube contents were mixed gently by inversion and centrifuged at the highest speed (12,000 g) for 3 min at 15 °C. Next, the aqueous layer was transferred to another tube, and one volume of chloroform/isoamyl alcohol (24:1) was added and centrifuged at the highest speed (12,000 g) for 3 min at 15 °C, and the supernatant was transferred to another fresh tube. RNA precipitation solution (2 µL glycogen and 1 mL polyethylene glycol) was added and centrifuged at 14,000 g for 60 min. The supernatant was discarded, and precipitated RNA was washed twice with ice-cold 75% ethanol and eluted in 50 µL low-EDTA TE buffer. Quality and quantity of the extracted RNA were checked by 1.5% agarose gel electrophoresis and spectrophotometry (Nanodrop, Thermo Fisher Scientific, Waltham, MA, USA), respectively. Genomic DNA contamination was removed by DNase treatment as per the manufacturer’s protocol (Thermo Fisher Scientific Waltham, MA, USA).

### 2.9. Gene Expression Analysis

The DNase-treated RNA served for first-strand cDNA synthesis using High-Capacity cDNA Reverse Transcription Kits (Applied Biosystems, Waltham, MA, USA) following the manufacturer’s instructions. Briefly, 500 ng of total RNA was mixed with 10 μL reaction mixture containing 3.2 μL nuclease-free water, 2 μL of 10X reaction buffer, 2.0 μL random primers, 0.8 μL of 25X dNTP mixes, 1.0 μL RNase inhibitor, and 1.0 μL MultiScribe^TM^ Reverse Transcriptase. Reactions were incubated for 10 min at 25 °C for initial annealing followed by extension for 2 h at 37 °C and final inactivation for 5 min at 85 °C. The resulting cDNA was used as a template for quantitative real-time PCR (qRT-PCR) using LightCycler^®^SYBR green fluorescence dye in a CFX96 real-time PCR detection system (Bio-Rad, Hercules, CA, USA). The reaction mixture consisted of 10 μL SYBR Green Master Mix, 0.8 μL (10 μM) each of forward and reverse gene-specific primers, 7.4 μL DDW, and 1 μL diluted template cDNA. The primers sequences were *xplA*f CCGAGTGGGCCAAACAGT *xplA*r TCCTCCTCGTCGAGTTCGAT, *gyrB*f GCCGAGGAGCAGGAACAG, and *gyrB*r TAGTGGTAGACGCGGGTCTTG [19]. The annealing temperature was 56 and 60 °C for *xplA* and *gyrB*, respectively. The qRT-PCR was then performed under the following conditions: 95 °C for 3 min followed by 40 cycles of 95 °C for 5 min, 56 °C for 20 s, and 72 °C for 20 s. Melting curve analysis was applied to all reactions to demonstrate primer specificity and amplification of a single product.. Standard curves for *xplA* with known copy numbers were generated by preparing a 10-fold dilution series of pJET plasmids carrying the respective target genes. The Ct (cycle threshold) and logarithm of the initial cDNA concentrations were plotted to calculate the slope. Standard curves were generated from at least four dilution points for each cDNA. The corresponding RT-qPCR efficiency E of one cycle in the exponential phase was calculated according to the equation E = 10(−1/slope) [34]. Expression of *xplA* was analyzed in 3 biological replicates; *gyrB* was used as an internal control gene to normalize gene expression. The relative change was calculated following [35]. Gene expression results were compared between experimental treatments using GraphPad Prism 9 and two-way ANOVA for the effects of treatment and time on fold expression of *xplA*.

## 3. Results

The study’s basic assumption was that perchlorate, chlorate, and nitrate, individually and as a mixture, would have either an adverse effect or no effect on RDX degradation by the three tested bacterial strains. The initial examination involved analyzing these strains’ growth and degradation activity in the presence of these environmentally relevant co-contaminants, found in polluted groundwater in Israel. In the presence of perchlorate, chlorate, nitrate, and their mixture, biomass yield after 96 h depended on the strain and treatment. Two-way RM (repeated measures) ANOVA revealed a significant effect of strain type on the biomass yield for all treatments (*p* < 0.0001). Pairwise comparison of biomass yield after 96 h of control treatment showed significantly higher yield for YH1 vs. T7 (*p* < 0.05) but not as compared to strain YY1. In the presence of perchlorate and chlorate, the biomass yield of YH1 (96 h) was significantly higher than those of the other two strains. This was not the case with the nitrate treatment. With the mixture of co-contaminants, the biomass yield of strain YY1 was higher than that of strain T7 (*p* < 0.001).

### 3.1. Growth Rates in the Presence of Environmentally Relevant Co-Contaminants

**Figure 1:**
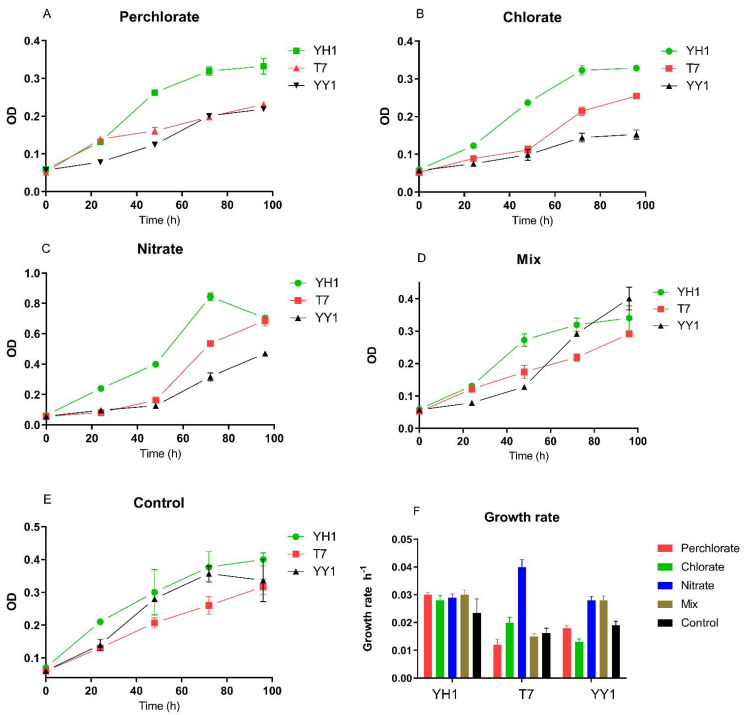
Effect of co-contaminants on the growth of three strains where RDX serves as the nitrogen source. (**A**) Perchlorate, (**B**) chlorate, (**C**) nitrate, (**D**) mix, (**E**) control, and (**F**) growth rate. The growth rate was derived from a nonlinear regression of the logarithmic growth phase. Triplicates were used in all treatments, and the results are average ± standard deviation.

Growth rates and doubling times were extracted from the growth curves by nonlinear regression of the log phase (Figure 1). We observed varied effects of perchlorate and chlorate on the growth of the strains when RDX was the sole nitrogen source. Perchlorate increased the growth rate of YH1 compared to the control, but this increase was not significant. For strain T7, the growth rate on nitrate was significantly higher than that for the control and the rest of the treatments. For strain YY1, the growth rate on nitrate and mixed contaminants was not significantly different but was higher than for the other treatments. Neither perchlorate nor chlorate were transformed in any of the experiments.

### 3.2. RDX Degradation Rates in the Presence of Environmentally Relevant Co-Contaminants

**Figure 2:**
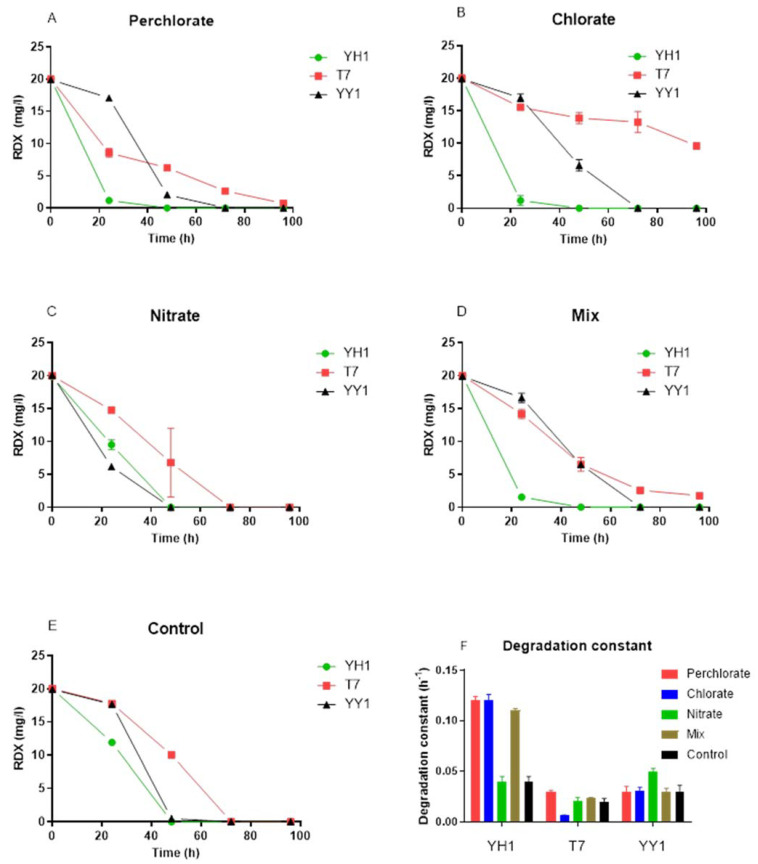
Effect of co-contaminants on RDX degradation rates of three strains of degraders. (**A**) Perchlorate, (**B**) chlorate, (**C**) nitrate, (**D**) mix, (**E**) control, and (**F**) growth constant. The degradation constant was obtained from a nonlinear regression of the first-order reaction. Triplicates were used in all treatments, and the results are average ± standard deviation.

A surprising observation was the acceleration of RDX degradation in the presence of co-contaminants (Figure 2). The effect was obvious in the presence of perchlorate, chlorate, and the contaminant mixture for strain YH1. Two-way RM ANOVA of the data revealed a significant effect of strain type on degradation constant for all treatments (*p* < 0.0001). For strain YH1, degradation constants were significantly higher for the perchlorate, chlorate, and mixed treatments than for the nitrate and control treatments. For strain T7, chlorate significantly decreased the degradation constant compared to the other treatments, but the degradation constants in the other treatments also differed. For YY1, only nitrate significantly increased degradation constants, whereas the other treatments were not significantly different.

### 3.3. Response of the Different Strains to Increasing Co-Contaminant Concentration

The dose response of the different strains was tested with a series of increasing concentrations of contaminants. It was hypothesized that the effect of increasing nitrate concentration on RDX degradation would be competitive; the strains would utilize nitrate and glucose, resulting in carbon limitation in the medium, and impairing RDX degradation. Furthermore, increasing concentrations of perchlorate and chlorate might affect bacterial growth and activity either via toxicity, or by affecting the salinity of the medium. To distinguish between these mechanisms, we included NaCl as a control.

**Figure 3:**
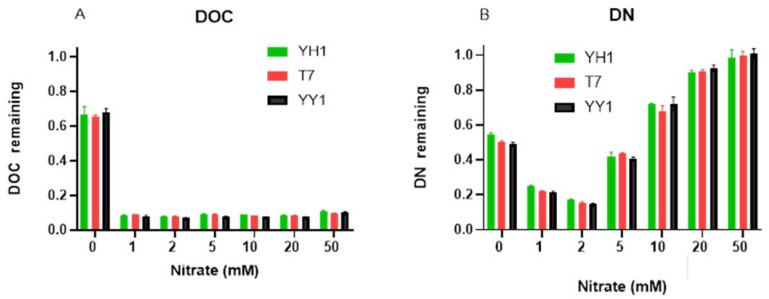
Residual DOC and DN (as a fraction of initial amounts) after 96 h incubation of the three strains with increasing nitrate concentrations. (**A**) DOC remained and (**B**) DN remained. All cultures initiated at an OD_600_ of 0.05 at time zero with 20 mg/L RDX and 1 g/L glucose. Triplicates were used in all treatments, and the results are average ± standard deviation.

Nitrate did not influence the extent of RDX degradation by strains YH1 and YY1, even at 50 mM after 96 h incubation. Strain T7 was affected by 20 and 50 mM nitrate, with 71.5 ± 2.8 and 61.2 ± 6.7% degradation (of the degradation in controls) after 96 h, respectively. Increasing nitrate concentration to 1 mM resulted in a 2-fold increase in biomass yield after 96 h compared to controls; however, further increases in nitrate concentration did not increase the yield for any of the strains (Figure S1). When evaluating the total organic carbon and total nitrogen remaining after 96 h (Figure 3), it appeared that even at 1 mM nitrate, 90% of the carbon was consumed. This suggests that the carbon in the medium becomes limiting, which may prevent further RDX degradation. However, this suggestion is probably only valid for strain T7, which did not degrade RDX completely. At the same time, concurrent RDX and nitrate utilization was apparent for all strains, but the results imply that from 5 to 50 mM nitrate, RDX is preferentially utilized. Overall, RDX degradation was not sensitive to nitrate competition, even under extreme concentrations.

**Figure 4:**
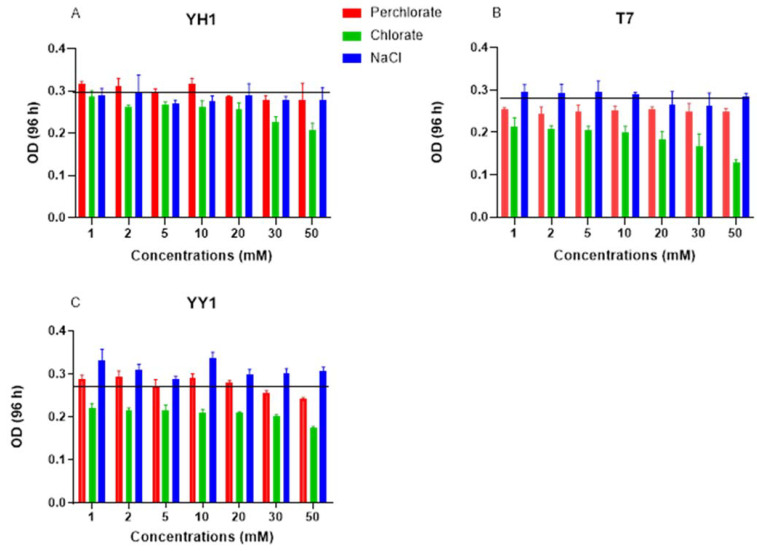
Biomass yield of the three RDX degrader strains at 96 h in the presence of increasing perchlorate, chlorate, and NaCl concentrations. Horizontal line represents the control yield. All cultures were initiated at an OD_600_ of 0.05 at time zero with 20 mg/L RDX and 1 g/L glucose. (**A**) Strain YH1, (**B**) strain T7, and (**C**) strain YY1. Results are average ± standard deviation of triplicate cultures.

Increasing perchlorate and chlorate concentrations did not affect the extent of RDX degradation by strains YH1 or YY1. For strain T7, 50 mM chlorate reduced RDX degradation to 60% (data not shown) at 96 h. The average control OD_600_ after 96 h was 0.3, 0.28, and 0.28 for strains YH1, T7, and YY1, respectively (Figure 4). For strain YH1, neither perchlorate nor NaCl influenced degradation or yield; a 33% decrease in yield was observed under 50 mM chlorate with no impact on degradation (Figure 4). Although neither perchlorate nor chlorate affected RDX degradation by strain YY1, they both reduced the biomass yield of this strain. At the same time, increased salinity did not affect either degradation or yield, implying that perchlorate and chlorate have a toxic effect on strain YY1. For strain T7, NaCl had no effect, and increasing concentrations of perchlorate did not change the culture OD. Increasing chlorate reduced biomass yield by up to 50% compared to the control at 50 mM (Figure 4).

### 3.4. Effect of Co-Contaminants on the Intrinsic Activity of XplA in Strains YH1, T7, and YY1

The effects of co-contaminants perchlorate and chlorate were also tested in a short-term assay. In the current study, chlorate and perchlorate unexpectedly enhanced the RDX degradation. Cells were grown in minimal medium containing RDX and perchlorate (800 mg/L) or chlorate (200 mg/L), and glucose as a carbon source. The cells were harvested and washed twice in minimal medium and transferred to fresh medium containing different concentrations of RDX (1–20 mg/L), and co-contaminants perchlorate (800 mg/L) or chlorate (200 mg/L), without carbon. The initial OD_600_ was 0.05 and the reaction time was 3 h.

**Figure 5:**
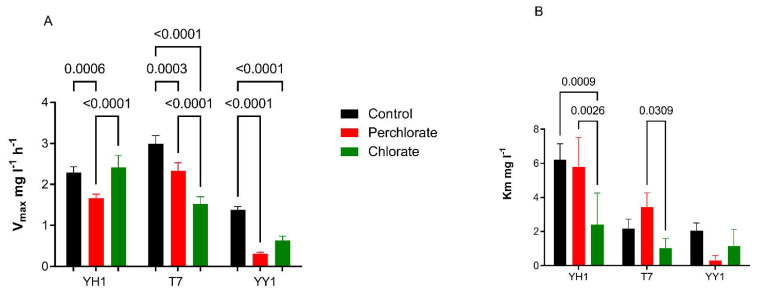
Effects of co-contaminants perchlorate and chlorate on RDX degradation of a resting cell suspension. Only significant *p*-values are presented. (**A**) Vmax, and (**B**) Km. The results are average ± standard deviation of triplicate cell suspensions for each RDX concentration.

The resting cell experiment aims to test the direct effect of the perchlorate and chlorate on the intrinsic activity of cytochrome P450, whereas in Figure 2, the effect was tested on growing cultures, so different degradation rates are to be expected. Nonlinear curve fitting produced the kinetic parameters of the Michaelis–Menten equation. As seen in Figure 5, there was no clear trend for the effects of perchlorate and chlorate on Vmax and Km. For example, Vmax in the presence of chlorate was significantly higher than in the presence of perchlorate for YH1, but not for T7 or YY1. For these latter two strains, Vmax of the control was considerably higher than with the chlorate and perchlorate treatments, suggesting their sensitivity to these oxyanions. In strain YH1, only chlorate significantly reduced Km relative to the control.

### 3.5. Expression of xplA in Co-Contaminant-Exposed Culture of Strain YH1

**Figure 6:**
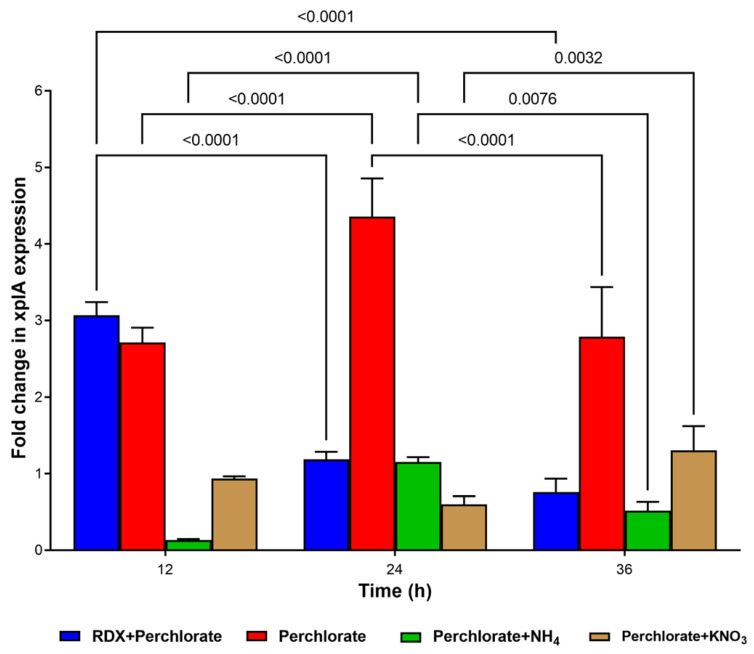
Effect of perchlorate alone or control treatments of perchlorate with ammonium and nitrate on *xplA* expression (fold change) during growth of strain YH1. Only significant *p*-values are presented.

An additional effect of co-contaminants on RDX degradation might be related to interference with *xplA* expression. Thus, we determined *xplA* expression in the presence of perchlorate, the main co-contaminant in the groundwater because no previous studies have reported RDX degradation and *xplA* expression in the presence of perchlorate under aerobic conditions. Strain YH1 was pre-grown in minimal medium supplemented with RDX (20 mg/L). Then, the following treatments were applied: RDX + perchlorate, perchlorate alone, perchlorate + ammonium, and perchlorate + nitrate, with glucose as the energy and carbon source. When the culture was spiked with perchlorate + RDX, *xplA* expression was enhanced about 3-fold at 12 h and then declined (Figure 6). A significant constant increase in expression level was seen between 12 and 24 h incubation with perchlorate alone; expression then declined significantly at 36 h.

The observation of peak *xplA* expression for RDX + perchlorate at 12 h followed by a significant decline agreed with our observation of complete degradation of RDX in the presence of perchlorate (Figure 2). As expected, ammonium and nitrate with perchlorate did not induce *xplA* expression.

No growth occurred in the treatment with perchlorate alone and, therefore, the question of whether XplA is active was investigated (Figure S2). XplA in the perchlorate-exposed cell suspension was active, albeit less than in the control (RDX), and at the same level as in the ammonium + perchlorate treatment (Figure S3). The lowest degradation activity was found for the perchlorate + nitrate treatment.

**Figure 7:**
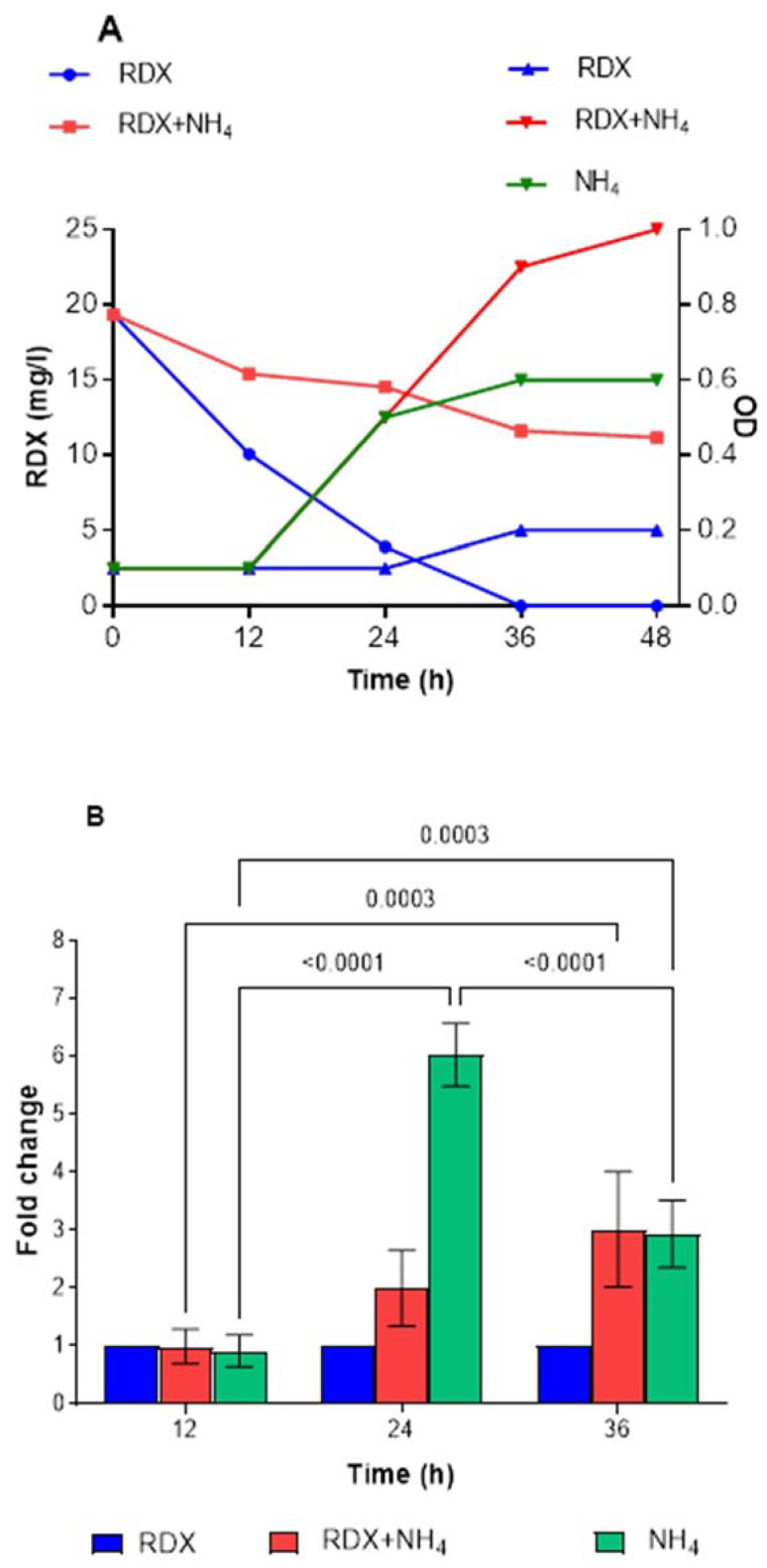
Degradation of RDX and growth of *Rhodococcus* strain YH1 in RDX, RDX + ammonium, and ammonium alone (**A**). Expression of *xplA* in strain YH1 in the presence of RDX, RDX + ammonium, and ammonium alone (**B**). Only significant *p*-values are shown.

To further examine the factors affecting *xplA* expression, we investigated the effect of ammonium. Only 30% degradation of RDX was observed with ammonium alone (Figure 7A), whereas complete degradation was achieved in the control after 36 h. The expression of *xplA* in this culture was significantly higher (~6- to 7-fold) after 24 and 36 h incubation (Figure 7B). However, in the treatment with both RDX and ammonium, the expression of *xplA* decreased compared to ammonia alone but was still significantly higher than the control, from 2-fold to 3-fold at 24 h and 36 h. The increased expression in the ammonium + RDX treatment was not translated to degradation activity, suggesting post-transcriptional regulation. Furthermore, when 24 h culture was harvested from the ammonium treatment, washed, and suspended in medium with RDX (no carbon), the degradation rate was 0.43 mg/L per h, 50% lower than for the RDX control (0.82 mg/L per h) (Figure S4).

## 4. Discussion

The polluted groundwater in the Israeli coastal aquifer contains high concentrations of perchlorate, chlorate, and nitrate at the plume center, which can affect RDX biodegradation. In contrast to anaerobic treatment, aerobic treatment of the polluted water followed by anaerobic perchlorate reduction can provide effluents without toxic reduced RDX intermediates. However, the co-contaminants’ potential to affect aerobic RDX degradation had never been evaluated. Our results reveal a significant strain-specific effect of the co-contaminants, with faster growth and faster degradation for strain YH1 compared to strains T7 and YY1, suggesting better adaptation.

In Israel’s polluted groundwater, nitrate is the primary inorganic nitrogen that can affect RDX degradation. In previous studies, the effect of nitrate on the growth and activities of the tested strains was examined. Strain YH1 degraded RDX in the presence of 1000 mg/L nitrate, albeit at a slower rate than in the control, suggesting competition with nitrogen [24]. Strain T7, however, did not degrade RDX in the presence of nitrate, but at the same time, it proliferated. With this strain, not all of the nitrate was consumed during incubation, implying that the culture had become carbon-limited [15]. Strain YY1 was not examined so far, and the current results suggest that its degradation activity is insensitive to nitrate (at the level tested). In comparison, strain 11Y cells grown under nitrogen-limiting conditions (450 μM NH_4_Cl, 450 μM KNO_3_, 450 μM KNO_2_, and 150 μM RDX) or incubated with no nitrogen, degraded RDX faster than cells grown under non-limiting nitrogen conditions (5 mM NH_4_Cl or KNO_3_) [19]. For this strain, external nitrogen, including nitrate, suppressed *xplA* expression, and this might be the mechanism affecting RDX degradation by strain YH1.

In contrast to nitrate, the effects of perchlorate or chlorate on RDX degradation are not known. It appears that in strains T7 and YY1, RDX degradation is insensitive to perchlorate, whereas strain T7 was negatively affected by chlorate. For strain YH1, the negative effect of nitrate on RDX degradation was offset by perchlorate and chlorate in the mixed treatment. These co-contaminants can impact either xplA directly or the expression of *xplA*. Our results disagreed with the observation of nitrate’s effect on RDX degradation by strain 11Y, where at 5 mM, it retarded the degradation rate [19]. Our combined observations of the growth and degradation kinetics of the three strains (Figure 1 and Figure 2) and the effect of nitrate concentrations indicate that they can also be effective in a high nitrate environment.

Our results suggest that chlorate is more toxic than perchlorate to all strains. Chlorate toxicity can result from its nonspecific reduction to chlorite by respiratory nitrate reductase [36,37]. In the current study, however, all strains could use nitrate as nitrogen through assimilatory nitrate reduction to ammonium. None contained the membrane-bound dissimilatory nitrate reductase (*narG*) [38].

The effect of the different co-contaminants on RDX degradation kinetics can be competitive, non-competitive, or uncompetitive, reflecting other interactions with the cytochrome P450 or enzyme-substrate complex. There was no effect for perchlorate, whereas for chlorate, the significant reduction in Vmax and Km were characteristic of uncompetitive inhibition. In the mixed treatment, the increase in Km was characteristic of a competitive effect; however, the rise in Vmax did not support this possibility. Chlorate is an inhibitor of various enzymes, including nitrite oxidoreductase and nitrate reductase, and acts as a competitive substrate for ATP-sulfurylase, a highly conserved prerequisite enzyme of the sulfate-reduction pathway [39,40,41,42]. Neither chlorate nor perchlorate is known to be an inhibitor of cytochrome P450 (XplA); however, chlorate may interfere with oxygen binding to the ferrous heme center of the enzyme. The observed increase in reaction velocity with the mixed co-contaminants was unusual, implying faster turnover of RDX by XplA. The addition of nitrate was responsible for this effect, yet the mechanism remains unclear in our study.

In other studies, expression of *xplA* in *Gordonia* sp. strain KTR9 was strongly induced by nitrogen limitation [18]. In experiments where ammonium (0.9 mM) in the culture medium was consumed, *xplA* expression increased up to 150-fold. *R. rhodochrous* 11Y expression of *xplA* (1.5-fold increase) and translation of XplA were also regulated by nitrogen limitation [19]. Resting cells of strain 11Y degraded RDX fastest after pre-growth on 250 μM RDX, and then 150 μM RDX, followed by pre-growth on 750 μM NH_4_Cl and then 450 μM NH_4_Cl. High nitrate and ammonium concentrations slowed down the activity of the resting cells. As for YH1, ammonium and nitrite strongly repressed cytochrome P450 (XplA) expression and RDX degradation in growing cultures. Nevertheless, ammonium itself did not suppress *xplA* transcription (Figure 7B); however, it seems that post-transcriptional regulation stops RDX degradation. Taken together, *xplA* expression is controlled by nitrogen limitation; its fold increase in the presence of perchlorate in YH1 is likely due to nitrogen limitation in the culture. Nevertheless, the activity of xplA in the 24 h cell suspension was lower (0.43 mg/L per h) than in the control (0.82 mg/L per h). This lower activity is likely due to the absence of reducing power (NADPH) in the non-growing culture (Figure S2). Our results indicate that there is no need for RDX as an inducer for active xplA, agreeing with the findings for strains 11Y and KTR9 [18,19].

## 5. Conclusions

We hypothesized that during ex-situ (pump and treat) bioremediation of RDX-contaminated groundwater, the presence of co-contaminants would hinder the aerobic degradation by *Rhodococcus* strains. Among the tested co-contaminants, we assumed that perchlorate and chlorate would be toxic, and nitrate would be a competitive inhibitor for degradation. The assumptions were tested for three RDX-degrading strains under different conditions. First, the effect of co-contaminants (at environmentally relevant concentrations) was found to be strain-specific, with YH1 being the least sensitive to them as its growth rate was not influenced. However, the first-order degradation constant was higher in the presence of perchlorate than in the control. Degradation and growth inhibition by chlorate were observed only for T7, raising a few questions: Is the effect concentration-dependent? Is the impact directly on cytochrome P450, or is it due to suppression of *xplA* expression?

With respect to nitrate, only T7 was affected by extremely high nitrate, whereas degradation by YH1 and YY1 was not affected. The amount of DOC remaining after 96 h suggests that T7 was carbon-limited. Nevertheless, most of the DN (nitrate) remained, and thus, nitrate may act directly on the enzyme or on *xplA* expression (in this strain). Similarly, high chlorate and perchlorate did not reduce the extent of RDX degradation. Still, chlorate reduced the biomass yield for all strains, particularly T7, indicating its toxicity, even at 1 mM. The mechanisms underlying this toxicity are not yet known. The strains were able to overcome this stress, as inferred from the absence of biomass decline with increased chlorate.

Expression of *xplA* increased in the presence of perchlorate alone after 24 h incubation of strain YH1, in contrast to the control treatments (perchlorate + RDX, perchlorate + nitrate, and perchlorate + ammonium). The xplA at this stage is active but at a lower rate than in the control, suggesting post-transcriptional regulation. In the treatment with perchlorate alone, the increased expression is likely due to nitrogen limitation. As expected, ammonium + nitrate repressed *xplA* expression. Although strong *xplA* expression was observed in ammonium-exposed cells, xplA activity was much lower than in the control, again indicating post-transcriptional regulation.

In conclusion, the examined strains demonstrated variable responses to the co-contaminants, with strain YH1 being the least affected and strain T7 the most. All three strains can be used for bioremediation of RDX-polluted groundwater containing an environmentally relevant mixture of co-contaminants.

## Data Availability

Data can be obtained from the corresponding author.

## Supplementary Materials

The following supporting information can be downloaded at: https://www.mdpi.com/article/10.3390/microorganisms10030663/s1, Figure S1: Biomass yield of the three strains after 96 h incubation with 20 mg/L RDX, 1 g/L glucose, and increasing nitrate concentrations. Figure S2: RDX degradation and growth of YH1 during the *xplA* expression experiment (Figure 6). Figure S3: XplA activity (degradation of RDX) in resting cell suspension of YH1 harvested after 24 h of incubation under the respective treatments. Figure S4: xplA activity (degradation of RDX) in resting cell suspension of YH1 harvested after 24 h of incubation under the respective treatments.
