# Supplementary material for: Effects of Perchlorate and Other Groundwater Inorganic Co-Contaminants on Aerobic RDX Degradation
Source: Microorganisms. 2022 Mar 20;10(3):663. doi: 10.3390/microorganisms10030663 (PMC8949498; doi:10.3390/microorganisms10030663)
Supplement: Supplementary file 1 [file microorganisms-10-00663-s001.zip › microorganisms-1627653-supplementary.pdf]

Effects of Perchlorate and Other Groundwater Inorganic Co-Contaminants on Aerobic RDX Degradation

Amit Yadav[1], Paula Istvan[1,2], Swati Gupta[1,3] and Zeev Ronen[1,*]

[1]Department of Environmental Hydrology and Microbiology, The Zuckerberg Institute for Water Research, The Jacob Blaustein Institutes for Desert Research, The Ben-Gurion University of the Negev, Sde Boker Campus 849900 Israel; yadava@post.bgu.ac.il, zeevrone@bgu.ac.il

[2]Center of Bioinformatics, University of Oslo and Cancer Registry, Oslo, N-0304 Norway; paula.istvan@gmail.com

[3]Department of Chemistry, Indian Institute of Technology Kanpur, Uttar Pradesh 208016, India; swati@iitk.ac.in

*Correspondence: Zeev Ronen zeevrone@bgu.ac.il Tel.: 972-8-6596895

Supplementary figures

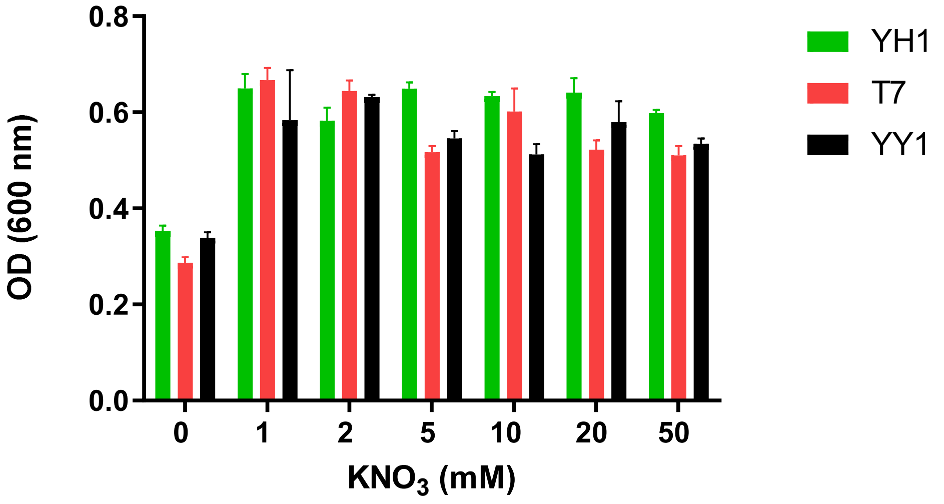

Figure S1. Biomass yield of the three strains after 96 h incubation with 20 mg/L RDX, 1 g/L glucose, and increasing nitrate concentrations.. Triplicates were used in all treatments, and the results are average ± standard deviation.

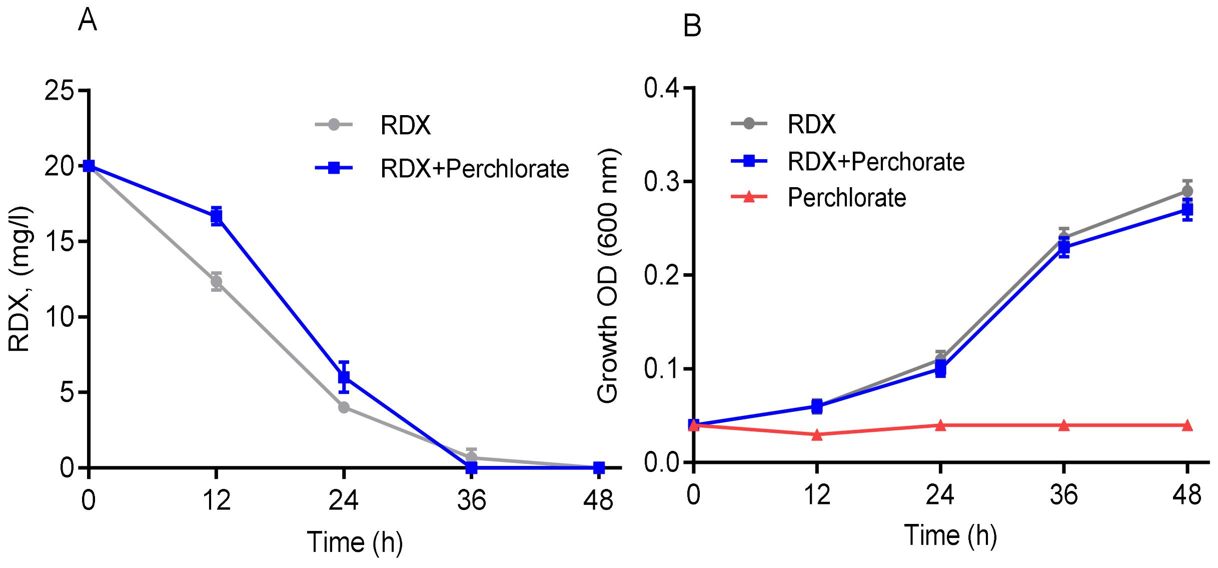

Figure S2. Degradation of RDX (A) and growth of strain YH1 (B) during the *xplA* expression experiment (Figure 6). Perchlorate was at a concentration of 800 mg/l, and the culture did not transform it. Triplicates were used in all treatments, and the results are average ± standard deviation.

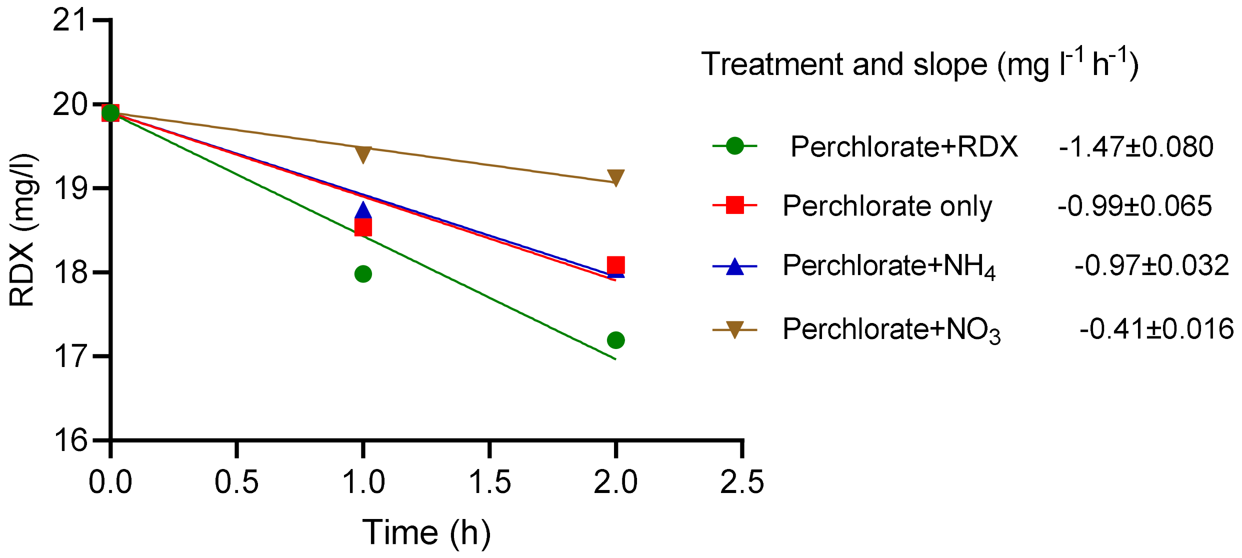

Figure S3. XplA activity (degradation of RDX) in resting cell suspension of YH1 harvested after 24h of incubation under the respective treatments. Triplicates were used in all treatments, and the results are average ± standard deviation.

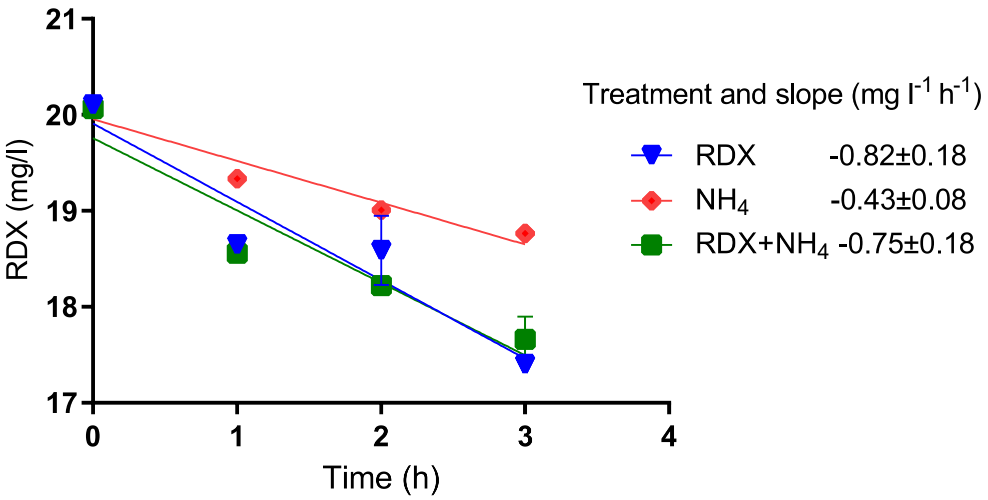

Figure S4. XplA activity (degradation of RDX) in resting cell suspension of YH1 harvested after 24h of incubation under the respective treatments. The results are average of triplicate suspensions.
